# Seasonal Temperature Changes Do Not Affect Cardiac Glucose Metabolism

**DOI:** 10.1155/2015/916016

**Published:** 2015-12-29

**Authors:** Jukka Schildt, Antti Loimaala, Eero Hippeläinen, Päivi Nikkinen, Aapo Ahonen

**Affiliations:** Department of Nuclear Medicine, HUS Medical Imaging Center, Helsinki University Central Hospital, Haartmaninkatu 4, 00029 Helsinki, Finland

## Abstract

FDG-PET/CT is widely used to diagnose cardiac inflammation such as cardiac sarcoidosis. Physiological myocardial FDG uptake often creates a problem when assessing the possible pathological glucose metabolism of the heart. Several factors, such as fasting, blood glucose, and hormone levels, influence normal myocardial glucose metabolism. The effect of outdoor temperature on myocardial FDG uptake has not been reported before. We retrospectively reviewed 29 cancer patients who underwent PET scans in warm summer months and again in cold winter months. We obtained myocardial, liver, and mediastinal standardized uptake values (SUVs) as well as quantitative cardiac heterogeneity and the myocardial FDG uptake pattern. We also compared age and body mass index to other variables. The mean myocardial FDG uptake showed no significant difference between summer and winter months. Average outdoor temperature did not correlate significantly with myocardial SUVmax in either summer or winter. The heterogeneity of myocardial FDG uptake did not differ significantly between seasons. Outdoor temperature seems to have no significant effect on myocardial FDG uptake or heterogeneity. Therefore, warming the patients prior to attending cardiac PET studies in order to reduce physiological myocardial FDG uptake seems to be unnecessary.

## 1. Introduction

The 2-[^18^F]fluoro-2-deoxy-D-glucose (FDG) positron emission tomography combined with computed tomography (PET-CT) is a widely used method for evaluating not only cancer, but also other disorders such as inflammation [1].

The heart can use various substrates as oxidative fuel and rapidly adapts its substrate utilisation to meet supply. Under nonfasting conditions, the major source for myocardial metabolism is fatty acids, but carbohydrates account for up to 30% of the metabolism [2]. Fasting with or without a high-fat, low-carbohydrate (HF-LCH) diet has been used to lower the physiological glucose metabolism in the myocardium, but there is no consensus over which protocol should be used in the diagnosis of cardiac sarcoidosis or in the assessment of cardiac viability [3]. Regulation of the myocardial carbohydrate metabolism is complex, and in addition to nutritional status, arterial substrates, and hormone levels, coronary flow and the inotropic state of the myocardium affect carbohydrate utilisation [4]. Results on the variability of intraindividual physiological myocardial FDG uptake over time remain controversial [5, 6], although in the clinical setting variability can be substantial.

Sarcoidosis is a multisystem granulomatous disease that can affect the myocardium in as many as 27% of cases [7]. Cardiac sarcoidosis is a potentially fatal condition and is associated with a shorter median survival time [8]. However, the diagnosis of cardiac sarcoidosis is demanding, and FDG-PET has proved to be a valuable addition to the diagnostic chain [9]. Thus, differentiating physiological from inflammatory myocardial FDG uptake is vital. An abnormal pattern of FDG uptake has been linked to myocardial pathologies; in cardiac sarcoidosis, for example, a focal uptake is considered an indicator of active disease [10]. In addition, greater heterogeneity in the myocardial SUV associates with cardiac sarcoidosis [11]. This tool could prove useful when interpreting cardiac FDG-PET scans but requires more verification. If FDG uptake in the normal myocardium varies greatly between scans, it would affect the overall heterogeneity of FDG uptake in the heart making interpretation more difficult. Seasonal variation in myocardial FDG uptake could be a factor to take into account when diagnosing cardiac sarcoidosis.

To our knowledge, no published studies have assessed the effect of outdoor temperature changes on myocardial FDG uptake or heterogeneity. In countries with a long winter, changes in temperature between seasons can be considerable. Thus, the aim of this study was to assess seasonal variability in myocardial FDG uptake.

## 2. Materials and Methods

### 2.1. Patients

The study, approved by the Helsinki University Hospital Institutional Review Board, comprised 29 consecutive patients referred to the Department of Nuclear Medicine at the Helsinki University Hospital for a PET-CT imaging as part of their routine diagnostic and/or treatment strategy during summer 2009 and again in the winter of 2009-2010. All patients referred to PET-CT had prior or suspected cancer. The indications for a PET study were Hodgkin's lymphoma (*n* = 9); non-Hodgkin's lymphoma (*n* = 2); head-and-neck cancer (*n* = 8); ventricular carcinoma (*n* = 2); rectosigmoid carcinoma (*n* = 2); cholangiocarcinoma (*n* = 2); esophageal carcinoma (*n* = 2); and pancreatic, renal, and lung cancer (*n* = 1). One patient was suspected of having carcinoma, but the final diagnosis was benign. The indication for the second PET study was either treatment response assessment or control of the previous findings. Of the 28 cancer patients, 6 underwent radiochemotherapy between the two studies, 12 underwent chemotherapy, and 10 received no treatment. None of the patients received radiotherapy to the lower mediastinum affecting myocardium. All 12 patients who received chemotherapy were treated with agents that are potentially cardiotoxic, such as doxorubicin, cisplatin, 5-fluorouracil, bevacizumab, and cyclophosphamide. However it has been previously shown that these oncological treatments do not affect the metabolic pattern of the myocardium or heterogeneity in the FDG distribution in the left ventricular walls [6]. All subjects were mobile outpatients and not in-house and represented a typical patient for a PET scan at our institution. We found BMI in our study population to be normal (range between 18,5 and 24,9 kg/m^2^), but a little lower than the average in Finland (21.5 ± 4.5, Finnish average 26.6) (National Institute for Health and Welfare, http://www.thl.fi/). Six subjects were under 16 years of age, 8 patients were underweight (BMI < 18.5 kg/m^2^), and 3 of these were under 16 years of age; only 1 was considered overweight (BMI > 30 kg/m^2^).

After the preliminary results, the patients were divided into two groups based on their myocardial SUVs. Patient Group 1 consisted of patients with a myocardial SUV higher in the winter than in the summer (*n* = 16), and Group 2, the opposite (*n* = 13).

### 2.2. PET-CT Imaging

The imaging protocol was identical for each individual in both studies. Patients were advised to fast for six hours prior to imaging. The blood glucose level was monitored, but the data was not obtainable from the first PET study as it was not routinely noted at the time. At the time of the second PET study the mean blood glucose level of the patients was 6,0 mmol/L (108 mg/dL) (SD ± 0,76, range 5,7–7,8 mmol/L). After an intravenous injection of ^18^F-FDG, the patients rested for 60 minutes in a semidarkened, quiet room. We used a Gemini PET/CT scanner (Philips Inc., Cleveland, OH, USA) to acquire the images. First, we obtained a CT surview (30 mA, 120 kVp). The patients were then scanned from the base of the skull to midthigh (50 mA, 120 kVp). We then acquired the PET images in a standard-manner 8 cm bed position at 1 min 30 sec per frame.

### 2.3. Data and Image Analysis

**Figure 1:**
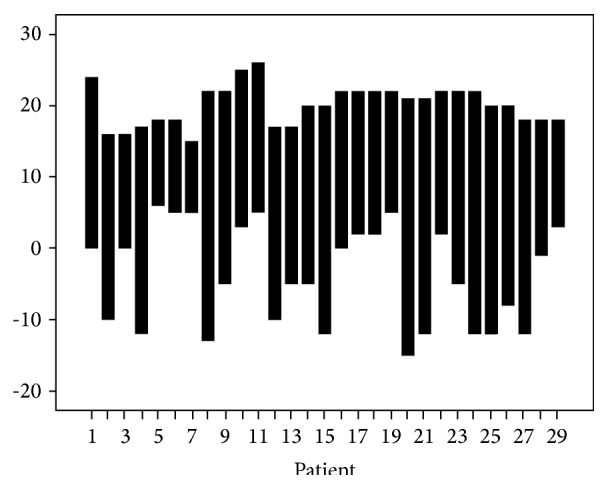
Temperature range (C) in summer and winter months for each study subject.

The PET studies in June, July, and August were considered a summer study and those in January, February, March, November, and December a winter study. We obtained average monthly and mean daily temperatures from the Finnish Meteorological Institute (http://www.ilmatieteenlaitos.fi/) (Figure 1) for Kaisaniemi weather station in Helsinki. The mean outside temperature change between the summer and winter studies was 24 (±7.1) degrees Celsius.

Two experienced physicians of nuclear medicine (JS, AA) evaluated the PET-CT images, which underwent quantitative analysis after hand-driven ROIs were drawn over the myocardium, mediastinum, and liver in order to obtain the SUVmax values for each area. The ROIs were drawn over the entire myocardium and partly over the mediastinum and liver indicating regions of diffuse uptake. We calculated SUVmax ratios for the myocardium/mediastinum and myocardium/liver.

**Figure 2:**
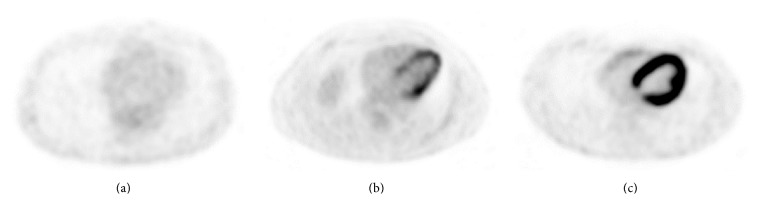
Different patterns of myocardial FDG distribution. (a) None, (b) moderate, and (c) intensive.

**Figure 3:**
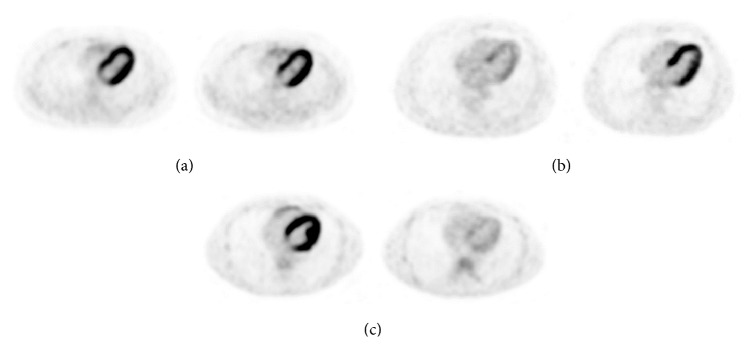
Examples of variations in myocardial FDG uptake over time. (a) “Intense” pattern both in summer and winter; (b) “none” pattern in summer, but “intense” in winter; (c) “intense” in summer and “none” in winter.

We then analysed the PET-CT images also visually and divided the patients into three categories (none/moderate/intensive) depending on the intensity and distribution of their myocardial FDG uptake (Figure 2). Changing from one category in one study to another category in the other study was considered a significant change in seasonal myocardial FDG uptake (Figure 3).

We assessed the heterogeneity of the myocardial FDG uptake according to the method previously described by Tahara et al. [11]. We used the Carimas software package developed at the Turku PET Centre for quantitation of cardiac PET studies to measure the SUVs of all of the 17 segments according to the statement of the American Heart Association [12, 13]. We calculated the coefficient of variation (CoV) of the myocardial SUV by dividing the standard deviation (SD) of the SUV by the average SUV.

### 2.4. Statistical Analysis

Baseline variables and patient characteristics appear as means, standard deviation (SD), and range. In order to detect a clinically significant difference in myocardial SUV and CoV between the two studies, SUV 2.8 and value of CoV 0.026 with an alpha value of 0.05 and a power of 80%, a total sample size of 29 patients was calculated to be sufficient. We compared the myocardial SUV and CoV in the summer months to those in the winter months and estimated associations between variables with Pearson's correlation analysis. We performed a stepwise regression analysis in order to detect significant associations between study variables and SUVs. An independent samples *t*-test was used to compare means between the two studies. The myocardial SUV data in both summer and winter was slightly skewed to the left, which means that logarithmic transformation is not mandatory. All other data was normally distributed.

## 3. Results

The characteristics of the study group are presented in Table 1. The average monthly temperature was 18.1°C in the summer months and −1.5°C in the winter months. The mean daily temperature in the summer months was 20.2 ± 2.8°C and −3.8 ± 7.0°C in the winter months, and the average temperature change between summer and winter was 24.0 (±7.1)°C. The injected FDG activity or the acquisition time did not differ between summer and winter months.  Table 2 summarises the variables.

**Figure 4:**
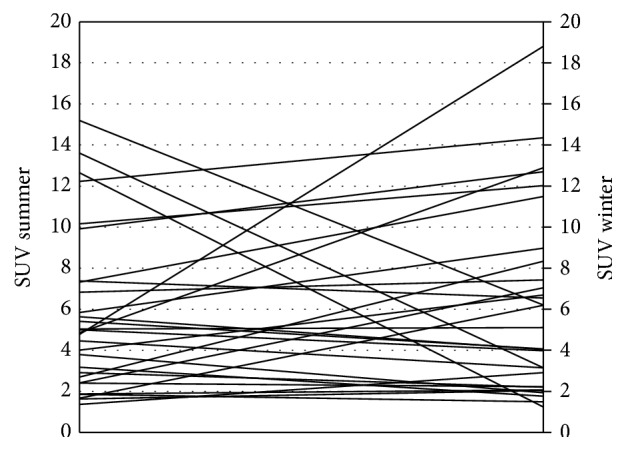
Myocardial SUVmax change from summer to winter for each subject.

We found no significant difference in the patients' myocardial FDG uptake between the summer and winter months (Figure 4). The difference in the myocardial SUVmax between the two studies, the myocardium/mediastinal SUVmax ratio, or the myocardium/liver SUVmax ratio was nonsignificant. The difference remained insignificant when looking only at patients over 16 years of age.

The average outdoor temperature did not correlate with the myocardial SUV in the summer or winter months (*r* = 0.18; *p* = 0.34 in summer; *r* = −0.3, *p* = 0.11 in winter). Additionally, the difference between summer and winter temperatures did not correlate with the difference in the myocardial SUV between the summer and winter months either (*r* = 0.12; *p* = 0.55).

The myocardial SUVmax in the winter months was higher than in the summer months in 16 (55%) of the 29 patients. The myocardial pattern was shifted upwards in 6 (38%) of these 16 patients, in 2 it shifted downwards, and in 8 it remained stable. Of the 29 patients, 13 (45%) had a higher myocardial SUVmax in the summer months; 7 (54%) of these 13 saw a downward shift in the myocardial pattern group, and 6 remained stable with no upward shift. Two very young (10–15 years old) subjects had active brown adipose tissue and a higher myocardial SUV in summer than in winter.

The patients' body mass index (BMI) had a positive correlation with the myocardial SUV in the summer (*r* = 0,39, *p* = 0.03) and especially in the winter (*r* = 0,45, *p* = 0.01) months.

**Figure 5:**
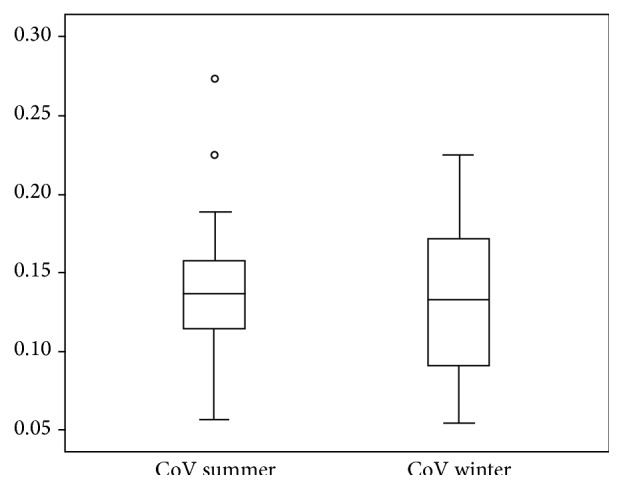
CoV of myocardial SUV in summer and in winter.

The heterogeneity of the myocardial SUV (CoV) mean was 0.14 (±0.05) in summer and 0.13 (±0.05) in winter; thus no significant difference in the heterogeneity between the summer and winter months (*p* = 0.527) was found (Figure 5). This was the case also when looking at the older patient population (>16 years) only.

## 4. Discussion

Our study focused on the seasonal variability of glucose metabolism of the myocardium. We compared intraindividual PET-CT studies performed at two time points: summer and winter. In addition to visually assessing the intensity and pattern of the myocardial FDG uptake, we used semiquantitative analysis with SUV values for the myocardium, mediastinum, and liver to obtain their ratios and calculated myocardial SUV heterogeneity. We found no significant difference in any of the parameters obtained between the summer and winter studies. According to our findings, warming the patients in order to diminish their physiological myocardial FDG uptake prior to a cardiac FDG-PET study seems unnecessary.

Certain patterns of FDG uptake in the myocardium are associated with various pathological conditions. Ishimaru et al. described different patterns of myocardial FDG uptake and concluded that focal uptake is characteristic of patients with cardiac sarcoidosis [10]. In a pictorial review, Lobert et al. illustrated varied pathological FDG uptake patterns associated with malignant conditions [14]. In our study, we found no significant seasonal variability in myocardial FDG patterns, so if the myocardial FDG pattern is considered pathological, the time of year of the PET scan may be irrelevant.

Tahara et al. assessed myocardial FDG uptake in 47 subjects. Of these, 24 had systemic sarcoidosis and 12 had cardiac involvement. The remaining 23 patients were control subjects, 8 of whom had dilated cardiomyopathy. The authors used the coefficient of variation of SUV as a marker for heterogeneity in the uptake. They found that the CoV was significantly greater in patients with cardiac sarcoidosis than in control or noncardiac sarcoidosis patients. They used a CoV cut-off value of 0.18, which provides high sensitivity and specificity for cardiac sarcoidosis (100% and 97%, resp.) [11]. In our study, we found no significant difference in the heterogeneity of myocardial FDG uptake between hot and cold months. Our findings indicate that outdoor temperature has no significant effect on myocardial glucose uptake and heterogeneity, so CoV can be used to assess cardiac sarcoidosis. This is an important finding, since the issue may be relevant to a large patient group; cardiac inflammatory processes are diagnosed worldwide, from North America to Northern and Central Europe, as well as in many Asian countries.

This almost automatic analysis method increases reliability of the results because it reduces interoperator variability, which can be substantial in visual analysis between operators or even within an operator.

The myocardial metabolism is complex and can adapt rapidly to changes in supply by shifting from one substrate to another. Many factors, such as nutrition and hormonal status, affect the mechanism of this process, which remains poorly understood [2]. Environmental conditions are major factors influencing the body and, consequently, the heart. Previous studies have addressed the influence of age, blood glucose level, fasting period, and dietary or pharmacological interventions on the myocardial glucose metabolism [3, 10, 15, 16]. There are ways to diminish myocardial glucose uptake and to turn myocardial metabolism towards fatty acid utilisation. Prolonged fasting for over 18 hours has proved effective [17], but this is a very demanding protocol for a patient with a serious illness such as cardiac sarcoidosis. Moreover, dietary protocols and intravenous heparin injections are widely used methods, but none of these have proved to be effective and consensus on proper imaging protocol is still lacking.

Intraindividual variation in the physiological metabolism of the myocardium could be a confounding factor when differentiating benign physiological from pathological glucose uptake in a PET scan with a glucose analog such as FDG. Whether myocardial FDG uptake varies significantly within individuals remains an open question. Khandani et al. found high reproducibility of cardiac FDG uptake on serial scans (intraclass correlation coefficient 0.77) in 47 patients, whereas Inglese et al. found significant variability over time in global myocardial FDG uptake and poor temporal reproducibility of myocardial SUV on serial PET scans (ICC 0.61) [5, 6].

To our knowledge, this is the first study to consider seasonal temperature changes as a factor affecting the glucose metabolism of the heart. The outside temperature between summer and winter varies greatly in Finland, exposing people to either very cold or very warm weather. The body adapts to these changes in ambient temperature in various ways; brown adipose tissue (BAT), for example, is known to be activated during the colder months [18], so common practice in the Nordic countries involves warming patients before scanning in order to eliminate any distracting uptake.

We found no significant difference in myocardial FDG uptake between warm summer months and cold winter months. Our study population comprised nearly as many subjects with a higher myocardial SUV in summer than in winter as subjects with a higher myocardial SUV in winter. The change in myocardial SUVs between summer and winter throughout the study population was quite dispersed, so we observed no consistent change. Outside temperature or changes in it therefore do not seem to be a significant factor influencing the metabolism of the heart.

Standard protocol requires that patients rest at least one hour before scanning, so in each case, they spend time indoors prior to their PET study. If the heart is able to adapt rapidly to warmer conditions, that is, within an hour, it could influence our findings.

## 5. Limitations

Because the patients in our study were all cancer patients, their fast lasted only 6 hours before the PET scan, contrary to the longer, 12-hour fast used when assessing cardiac inflammation such as CS. This is a minor limitation, as the purpose of our study was to investigate intraindividual physiological variation in myocardial FDG uptake.

We did not control the time each subject spent outdoors before their scans. Our study aimed to compare scans taken in different seasons and the effect of a long time exposure to different ambient temperatures on myocardial FDG uptake. All of the subjects in this study were mobile outpatients and not in-house patients representative of a typical patient for a PET scan at our institution.

We could not evaluate the possible effect of blood glucose levels on the seasonal variation in myocardial FDG uptake, since the blood glucose levels were not routinely noted at the time of the first scans. At the time of the second scan, however, we found no significant correlation between the blood glucose levels and the myocardial SUVs.

## 6. Conclusion

In conclusion, our study shows that outside temperature changes between seasons seem to have no significant effect on myocardial FDG uptake. In addition, seasonal variance in outside temperature does not affect heterogeneity of myocardial FDG uptake. The glucose metabolism of the myocardium does not react to seasonal temperature changes in the same way as brown adipose tissue does, so such temperature changes should not be a confounding factor in myocardial FDG-PET studies, such as when a diagnosis of cardiac sarcoidosis is in question. Warming the patients prior to cardiac FDG-PET studies to lower the physiological myocardial FDG uptake seems to be unnecessary.

## Figures and Tables

**Table 1:** Characteristics of patients.

Number of patients	29
Male/female	19/10
Age mean ± SD (range)	47.9 ± 21.4 (10–72)
BMI mean ± SD (range)	21.5 ± 4.5 (14.9–34.0)

**Table 2:** Temperatures, SUVs, and coefficients of variation for the two scans.

	First scan	Second scan	*p* value
	Mean (±SD)	Mean (±SD)	
Activity mean (MBq)	339 (58.5)	340 (57.0)	0.828
Acquisition (min)	62,9 (8.72)	60.5 (5.51)	0.234
Temperature mean (range) (°C)	20.2 (15–26)	−3.8 (−15–6)	
SUVmyo	5.7 (±3.9)	6.2 (±4.6)	0.586
SUVmed	1.8 (±0.4)	1.8 (±0.2)	0.373
SUVliv	2.5 (±0.5)	2.6 (±0.5)	0.460
SUVmyo/med	3.2 (±2.2)	3.6 (±2.7)	0.478
SUVmyo/liv	2.4 (±1.8)	2.5 (±1.9)	0.796
Coefficient of variation	0.139 (±0.046)	0.134 (±0.050)	0.527
